# Mobile Health Apps for Breast Cancer: Content Analysis and Quality Assessment

**DOI:** 10.2196/43522

**Published:** 2023-02-23

**Authors:** Seongwoo Yang, Cam Nhung Bui, Kyounghoon Park

**Affiliations:** 1 HERINGS, The Institute of Advanced Clinical & Biomedical Research Seoul Republic of Korea; 2 Department of Digital Health Samsung Advanced Institute for Health Sciences & Technology Sungkyunkwan University Seoul Republic of Korea

**Keywords:** app, breast cancer, quality assessment, mobile health, mHealth, digital health, digital health intervention, cancer management, tablet, prevention, survivor, peer-support

## Abstract

**Background:**

The number of mobile health apps is rapidly increasing. This means that consumers are faced with a bewildering array of choices, and finding the benefit of such apps may be challenging. The significant international burden of breast cancer (BC) and the potential of mobile health apps to improve medical and public health practices mean that such apps will likely be important because of their functionalities in daily life. As the app market has grown exponentially, several review studies have scrutinized cancer- or BC-related apps. However, those reviews concentrated on the availability of the apps and relied on user ratings to decide on app quality. To minimize subjectivity in quality assessment, quantitative methods to assess BC-related apps are required.

**Objective:**

The purpose of this study is to analyze the content and quality of BC-related apps to provide useful information for end users and clinicians.

**Methods:**

Based on a stepwise systematic approach, we analyzed apps related to BC, including those related to prevention, detection, treatment, and survivor support. We used the keywords “breast cancer” in English and Korean to identify commercially available apps in the Google Play and App Store. The apps were then independently evaluated by 2 investigators to determine their eligibility for inclusion. The content and quality of the apps were analyzed using objective frameworks and the Mobile App Rating Scale (MARS), respectively.

**Results:**

The initial search identified 1148 apps, 69 (6%) of which were included. Most BC-related apps provided information, and some recorded patient-generated health data, provided psychological support, and assisted with medication management. The Kendall coefficient of concordance between the raters was 0.91 (*P*<.001). The mean MARS score (range: 1-5) of the apps was 3.31 (SD 0.67; range: 1.94-4.53). Among the 5 individual dimensions, functionality had the highest mean score (4.37, SD 0.42) followed by aesthetics (3.74, SD 1.14). Apps that only provided information on BC prevention or management of its risk factors had lower MARS scores than those that recorded medical data or patient-generated health data. Apps that were developed >2 years ago, or by individuals, had significantly lower MARS scores compared to other apps (*P*<.001).

**Conclusions:**

The quality of BC-related apps was generally acceptable according to the MARS, but the gaps between the highest- and lowest-rated apps were large. In addition, apps using personalized data were of higher quality than those merely giving related information, especially after treatment in the cancer care continuum. We also found that apps that had been updated within 1 year and developed by private companies had higher MARS scores. This may imply that there are criteria for end users and clinicians to help choose the right apps for better clinical outcomes.

## Introduction

With the increasing use of mobile apps since 2010, the number of mobile health–related (mHealth) apps has also risen [1]. According to a 2021 IQVIA report, there is a growing number of digital health care apps, with >350,000 apps related to health and fitness or medical categories available in the App Store and Google Play [2]. In 2020, a national survey in the United States found that more than half of all mobile phone users had downloaded health-related apps, and among them, two-thirds felt that such apps helped improve their health [3]. The key features of these health-related apps are maintaining a medication log, monitoring side effects, and scheduling follow-up appointments [4]. In addition, disease-specific apps may empower patients to promote self-efficacy, and self-care behavior in daily life, via information-technology services such as educational, patient-to-patient, and electronic patient-reported outcome services [5]. Therefore, maximizing the use of these advanced smartphone functions has paved the way for the delivery of diverse health care services [6]. In this respect, clinicians may want to employ useful apps to their patients and monitor their effect on patient outcomes [7]. However, to encourage health care providers to exploit them with confidence, there is a need for unbiased and scientific assessment of mHealth apps [6]. In addition, studies on mHealth have found that consumers are faced with a “bewildering array” of such apps because of the difficulty in discerning app quality, despite their beneficial functions [8].

Furthermore, since most public reviews of apps are based on individuals’ own subjective experience, more scientific and systematic assessment of mHealth apps is important [6]. Therefore, methods for the systematic search and analysis of apps have been developed [9]. Using these methods, several studies have evaluated apps concerning general cancer care [4,10], specific cancer types (eg, prostate cancer [11,12]), skin monitoring and melanoma detection [13,14], and medication compliance [15,16].

Even though breast cancer (BC) is one of the most prevalent cancers, especially in high-income countries, and is the leading cause of death in women in most low-income and many middle-income countries [17,18], morbidity and mortality can be reduced by promoting exercise, a healthy diet, adequate access to screening services, treatment, and care management [19,20]. However, a lack of information and support has prevented many women with BC from engaging in healthier behaviors during their cancer care [17,19,20]. Additionally, increased rates of early diagnosis and treatment [21] and a better prognosis and survival than other cancer types have resulted in unmet supportive-care needs for BC survivors [22,23]. In this sense, mHealth interventions can serve as promising platforms to enhance preventive and postdiagnosis behavior change with a clear goal setting and adherence to relevant theories [24]. Therefore, in terms of the international burden of BC and the potential of mHealth apps to improve medical and public health practices, the use of BC-related apps across the cancer care continuum (CCC) is important because of their functionalities in daily life [25].

According to a systematic review, in their nascent stage, BC-specific apps focused mainly on resources for BC awareness, screening, diagnosis, and treatment, so there is a lack of evidence on their utility, effectiveness, and safety [4]. Another systematic review analyzed all BC-related apps for their content and adherence to design standards outlined by the Institute of Medicine, as well as the relationships between their content, user ratings, and price [26]. The review found that mHealth apps have not met their potential for consumer engagement with evidence-based information, and BC-specific apps represented a limited spectrum on the cancer continuum [26]. Another systematic review that targeted BC survivorship and self-management pointed out that very few relevant resources were available in the apps considered [6], despite their utility in alleviating the burden and costs for BC survivors [27]. In sum, all these studies scrutinized cancer- or BC-related apps, but their focus was on mobile app availability, and they relied on user ratings to decide app quality. Therefore, to minimize subjectivity in quality assessment, quantitative methods to assess BC-related apps are required.

mHealth apps related to BC have different contents and features; therefore, the primary goal of this study was to perform a detailed evaluation of each element. In particular, patients with BC receive long-term care; they need proper information and direction outside the hospital setting through mobile apps. Therefore, the secondary goal of the study was to analyze the quality of BC-related apps to provide useful information for users and clinicians.

## Methods

### Overview of Mobile Apps

This stepwise systematic approach evaluated BC-related smartphone apps available on the Android and iOS platforms that had features related to cancer prevention, detection, treatment, and the provision of survivor support. We used the keywords “breast cancer” in English and Korean to identify commercially available apps in Google Play and the App Store, using accounts in both the United States and South Korea. The search was conducted on July 1, 2022, using the app search engine AppAgg, a mobile app metadata resource that was also used in a related study [28]. We recorded each app’s title, developer, final update, description, price, and website address.

### Selection Criteria

This study included English and Korean BC-related apps for women who are at risk for BC across the life stages in the relevant app categories (health and fitness, medical, social, and lifestyle) that had been updated within the previous 3 years (July 2019-July 2022) and were available free of charge. We excluded apps that did not function correctly (eg, unreadable text or a blank screen), those that merely provided lists of conditions, and those intended for medical students that used self-made flashcards. In addition, we excluded apps that were developed with specific target users in mind (eg, those for health care professionals or children), to prompt a donation, or for trial recruitment. The eligible apps for Android and iOS were installed and alternately tested by each reviewer on a Samsung Galaxy S21 (Android version 11.0; Google LLC) and an iPhone 11 (iOS version 15.5; Apple Inc), respectively.

### Content Analysis

The apps were independently evaluated by 2 investigators (SY and CNB). We recorded each app’s title, platform, developer, category, date of latest update, language, and description. The content and functions of the selected apps were classified using the CCC, which has been used since the mid-1970s to describe the various stages of cancer in terms of etiology, prevention, detection, diagnosis, treatment, and survivorship [29,30]. Although the CCC categories are not discrete due to their oversimplified nature, they provide useful labels based on the development of cancer biology. We adopted the coding scheme proposed by Charbonneau et al [10], which redefined the following 7 categories of cancer apps identified by Bender et al [4]: educational, fundraising, prevention, early detection, disease and treatment information, disease management, and support. By integrating these concepts with the CCC stages, we created new categories that covered the app features identified in previous studies (Multimedia Appendix 1). We assumed that the functions and content of BC-related apps would fit into these categories.

### Quality Assessment

The Mobile App Rating Scale (MARS) was used to evaluate the quality of the selected apps. This scale is a commonly used and validated tool that evaluates the following 5 dimensions of mobile apps: engagement, functionality, aesthetics, information, and subjective quality [31,32]. The apps are scored on a 5-point scale (1=inadequate; 2=poor; 3=acceptable; 4=good; and 5=excellent). Two blinded reviewers evaluated the apps separately without sharing detailed information; the Kendall coefficient of concordance was calculated to evaluate the agreement between them [33]. The apps were evaluated based on the MARS scores (total and dimension), and the mean scores of the 2 raters were calculated. We further analyzed differences in MARS scores by content based on the CCC and the number of years since the last update. We also classified the apps by the type of developer such as individual, commercial (including private companies or for-profit organizations), or public institutions (including nongovernmental organizations, hospitals, government agencies, or universities). For multiple comparisons of the numbers of years since last updated and developer types, analysis of variance and the Tukey honestly significant difference test were performed. Statistical analyses were performed using R software (version 4.1.0; R Foundation for Statistical Computing).

### Ethical Considerations

Since this study contains no primary data obtained from any experiment on human subjects, ethics approval was not required.

## Results

### App Identification

**Figure 1:**
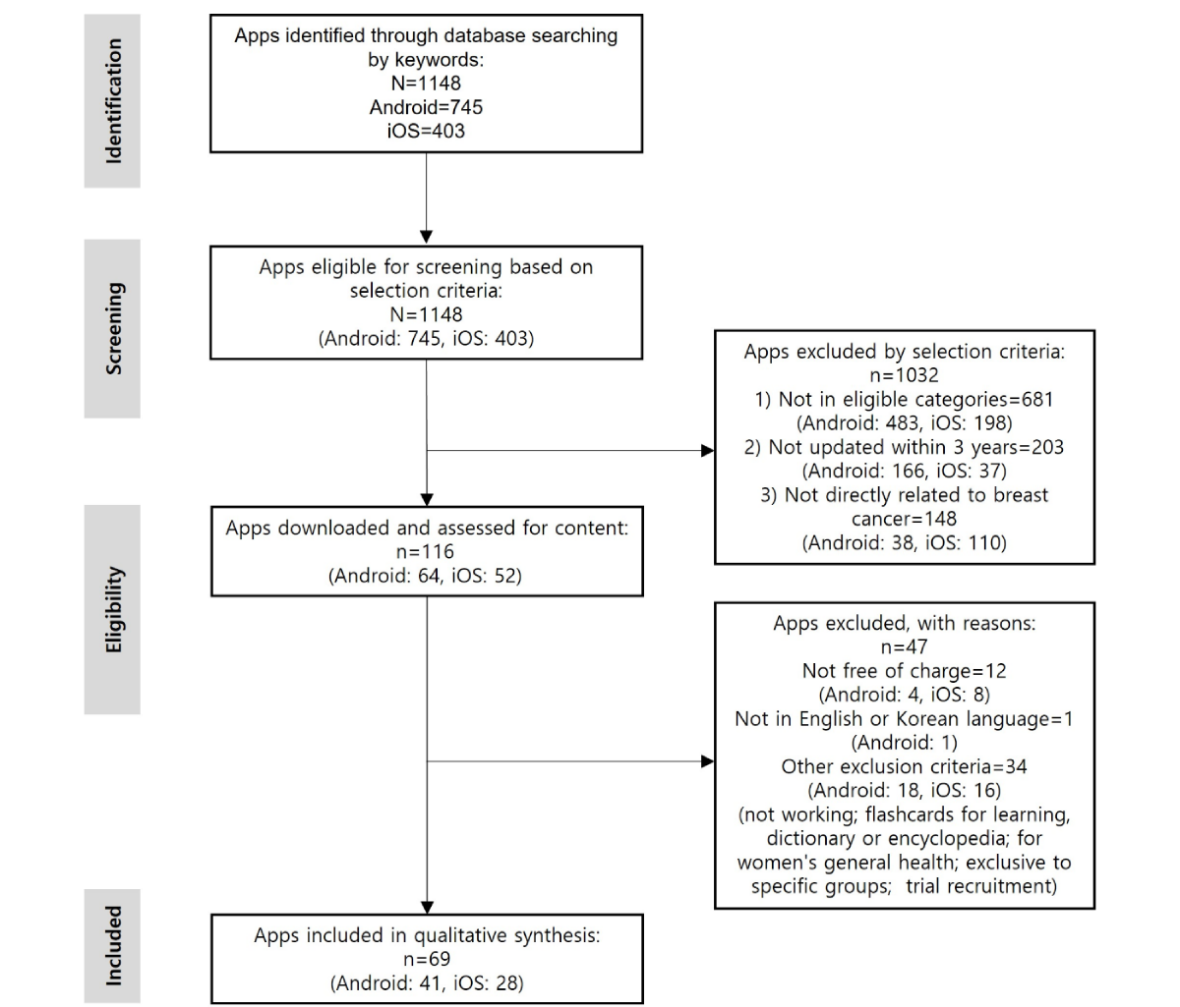
Flow diagram for the selection of breast cancer mobile health apps.

In total, 1148 apps identified through the database search were reviewed for eligibility for this study. After applying our exclusion screening criteria (eligible categories, updates, and relevance), 116 apps were downloaded and assessed in terms of content, price, language, and other criteria. Finally, 69 apps (n=41, 59% Android apps and n=28, 41% iOS apps) were included and subjected to content and quality assessment (see Figure 1 for the app-selection process and Multimedia Appendix 2 for the list of 69 BC-related apps included).

### General Characteristics of the Included Apps

Of the 69 apps selected, 41 (59%) and 28 (41%) were available only on Android Google Play and the Apple App Store, respectively (Table 1), while 15 (22%) were available on both platforms. Most apps were included in the Health and Fitness category (n=48, 70%), had been updated within the previous year (n=46, 67%), were developed by a commercial organization (n=43, 62%), and were available in English (n=62, 70%).

**Table 1:** General characteristics of the 69 included apps.

Characteristics		Values, n (%)
**Platform**		
	Android	41 (59.4)
	iOS	28 (40.6)
	Android and iOS	15 (21.7)
**Category**		
	Health and fitness	48 (69.6)
	Medicine	15 (21.7)
	Lifestyle	2 (2.9)
	Social networking	4 (5.8)
**Updated within**		
	1 year	46 (66.7)
	2 years	8 (11.6)
	3 years	15 (21.7)
**Developer**		
	Individual	8 (11.6)
	Commercial organization^a^	43 (62.3)
	Public institution^b^	18 (26.1)
**Language**		
	English	62 (89.9)
	Korean	2 (2.9)
	English and Korean	5 (7.2)

^a^Such as private company or for-profit organization.

^b^Such as nongovernmental organization, hospital, government agency, or university.

### Content Analysis

Table 2 shows the contents of the BC-related apps according to category and platform (Android and iOS). Their most common function was to provide information related to early detection of BC (n=34, 49%), followed by information concerning the risk factors and biological processes of BC at the prevention stage (n=28, 41%). The next most common content was information about symptoms, treatments, new advancements in BC treatment, and side effects related to BC after the treatment stage (n=27, 39%); education for lifestyle modification (n=23, 33%), and education for facts and knowledge related to BC (n=22, 32%). The apps also recorded patient-generated health data (PGHD), including tracking patients’ and survivors’ exercise, sleep, diet, and symptoms (n=19, 28%); provided psychological support (n=17, 25%); and assisted in medication management (n=16, 23%). However, content related to PGHD and medication management was more likely to be included in apps for iOS than in those for Android. The content of BC-related apps was also analyzed according to the number of years since the last update and the developer type (Multimedia Appendix 3). We found that 46 (67%) apps had been updated in the previous year, and 43 (62%) were developed by commercial organizations. Additionally, the apps that were outdated or had been developed by individuals mainly provided information or education regarding prevention or treatment. By contrast, those that had recently been updated and those that were developed by commercial organizations provided diverse content, including guidance for early detection, recording of PGHD, facilitation of medication management, and promotion of lifestyle modifications. Details of the apps’ contents are provided in Multimedia Appendix 4.

**Table 2:** Content analyses of the 69 breast cancer (BC)–related mobile health apps.

Cancer control continuum and content		Android (n=41), n (%)	iOS (n=28), n (%)	Total (n=69), n (%)
**Etiology and prevention**				
	Information on BC	19 (46.3)	9 (32.1)	28 (40.6)
	Risk prediction	8 (19.5)	2 (7.1)	10 (14.5)
	Education for prevention and risk factors for BC	16 (39.0)	6 (21.4)	22 (31.9)
**Detection**				
	Guidance for early detection	21 (51.2)	13 (46.4)	34 (49.3)
	Connection to professionals	5 (12.2)	5 (17.9)	10 (14.5)
**Diagnosis and treatment**				
	Information on BC treatment	17 (41.5)	10 (35.7)	27 (39.1)
	PGHD^a^	8 (19.5)	11 (39.3)	19 (27.5)
	Medical records	4 (9.8)	2 (7.1)	6 (8.7)
	Medication management	7 (17.1)	9 (32.1)	16 (23.2)
	Consultation by a physician	3 (7.3)	1 (3.6)	4 (5.8)
	Tracking appointments	4 (9.8)	5 (17.9)	9 (13.0)
	Participation in decision-making	5 (12.2)	4 (14.3)	9 (13.0)
**Survivorship**				
	Information on posttreatment care and prevention of recurrence	8 (19.5)	6 (21.4)	14 (20.3)
	Education for lifestyle modification	15 (36.6)	7 (25.0)	23 (33.3)
	Consultation with an expert	6 (14.6)	4 (14.3)	10 (14.5)
	Psychological support	7 (17.1)	10 (35.7)	17 (24.6)
	Community	7 (17.1)	6 (21.4)	13 (18.8)
	Sharing information with family and caregivers	5 (12.2)	2 (7.1)	7 (10.1)
	Fundraising	3 (7.3)	5 (17.9)	8 (11.6)

^a^PGHD: patient-generated health data.

### Quality Assessment of the Apps

**Figure 2:**
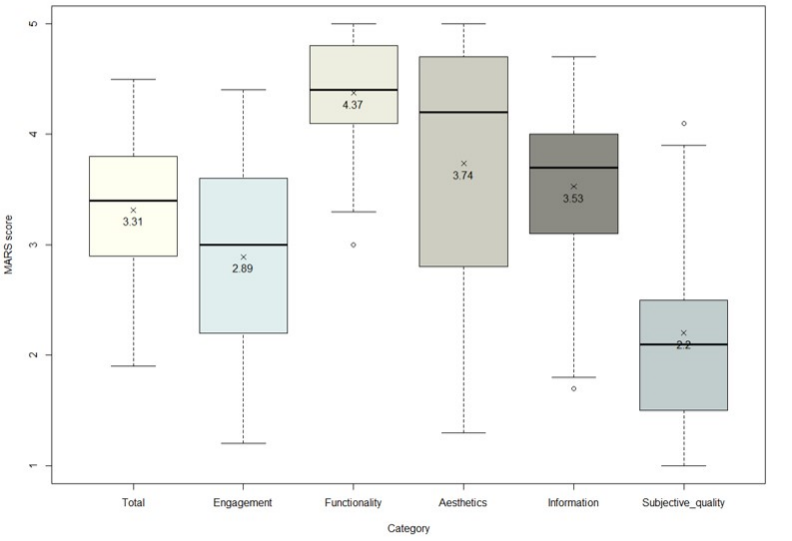
App scores by the dimension of the Mobile App Rating Scale (MARS; n=69).

The 69 apps were evaluated by 2 raters using the MARS (Figure 2). The Kendall coefficient of concordance was 0.91 (*P*<.001), indicating a good agreement between the raters; disagreements between them were resolved by consensus. The mean total score (1-5) of the included apps was 3.31 (SD 0.67; range: 1.94-4.53). Among the MARS dimensions, functionality had the highest mean score (4.37, SD 0.42), followed by aesthetics (3.74, SD 1.14), information (3.53, SD 0.69), engagement (2.89, SD 0.92), and subjective quality (2.20, SD 0.79). The dimension scores of the apps varied widely, indicating large variations in app quality. The MARS scores of the included apps are presented in Multimedia Appendix 5. Of the 69 apps included, *OncoPower* (Android: 4.0, iOS: 4.2), *Outcomes4Me Breast Cancer Care* (Android: 4.4, iOS: 4.5), *OWise – Breast Cancer Support* (Android: 4.2, iOS: 4.1), and *War On Cancer* (Android: 4.0, iOS: 4.0) had the highest scores. Among the MARS dimensions, they scored especially highly in functionality and aesthetics (Multimedia Appendix 5).

**Figure 3:**
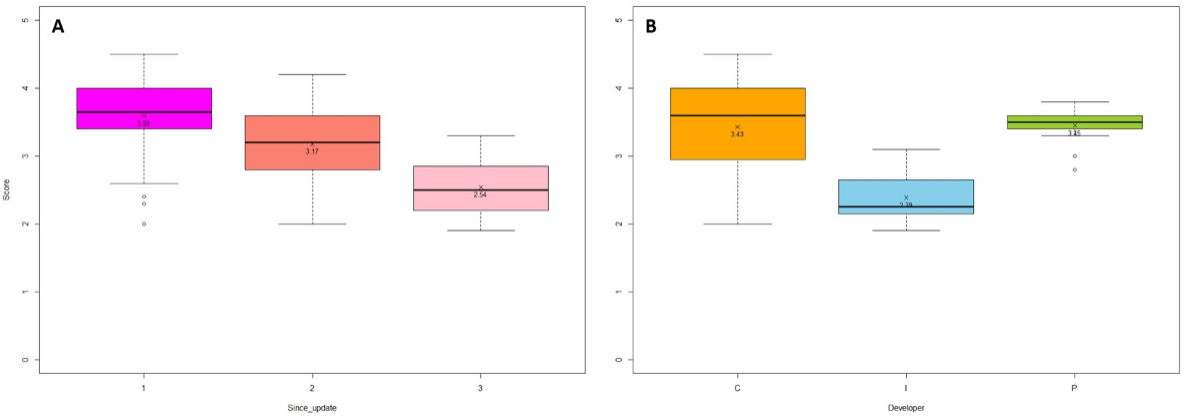
Mobile App Rating Scale (MARS) total scores by (A) years since apps were last updated and (B) type of developer. C: commercial; I: individual; P: public institution.

The MARS scores were analyzed based on the number of years since the app was last updated and the developer type (Figure 3). Tukey test showed that the mean scores for apps that had been updated within the previous 1 (3.59, SD 0.53) or 2 (3.17, SD 0.68) years were not statistically different. However, outdated apps (ie, those that had been updated >2 years previously) had a significantly lower mean MARS score (2.54, SD 0.41) compared to the other apps (*P*<.001). Apps developed by individuals had a significantly lower mean MARS score (2.39, SD 0.41) than those developed by commercial organizations (3.43, SD 0.70) and public institutions (3.45, SD 0.28; *P*<.001).

### Quality Assessment of the Apps’ Content

**Figure 4:**
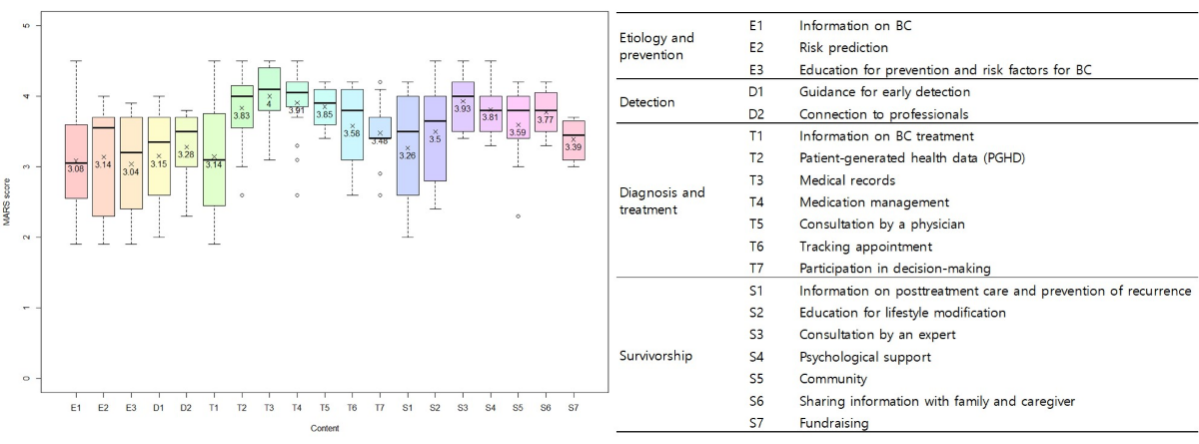
Content quality assessment (n=69). D: detection; E: etiology; MARS: Mobile App Rating Scale; S: survivorship; T: treatment.

The quality of the content of the included apps was assessed using the MARS (Figure 4). Apps that tracked treatment records had the highest mean MARS score (T3; 4.0, SD 0.51), followed by those that facilitated consultations with experts (S3; 3.93, SD 0.38), medication management (T4; 3.91, SD 0.5), and consultations with physicians (T5; 3.85, SD 0.34). The mean MARS score was higher for apps that involved recording PGHD (T2; 3.83, SD 0.5), provided psychological support (S4; 3.81, SD 0.37), and shared information with family members or caregivers (S6; 3.77, SD 0.35) or the community (S5; 3.59, SD 0.53). Apps that only provided information on BC prevention or risk factors had significantly lower MARS scores than the other apps.

## Discussion

### Principal Findings

We evaluated the contents and quality of BC-related mobile apps using the MARS. We organized the CCC using the results of previous studies to establish specific definitions for the content of mobile apps for women. Our results are based on 69 BC-related mHealth apps. The most frequent content of the apps was provision of information related to early detection of BC (n=34, 49%), followed by information regarding risk factors and biological processes of BC at the prevention stage (n=28, 41%). The next most frequent content was information about symptoms, treatments, new advancements in BC treatment, and side effects related to BC after the treatment stage (n=27, 39%). Education regarding lifestyle modification (n=23, 33%), prevention and risk factors of BC (n=22, 32%), and PGHD (n=19, 28%) were employed as content in BC-related apps. A total of 46 (68%) apps had been updated in the previous year, and 43 (62%) were developed by private companies.

The mean MARS score of the included apps was 3.31 out of 5, which was higher than the “acceptable” MARS score of 3 [31]. However, there was a significant gap between the highest- and lowest-rated apps (score range: 1.94-4.53). Functionality had the highest mean score (4.37, SD 0.42) among the MARS dimensions, and outdated apps (mean 2.54, SD 0.41) and apps developed by individuals (mean 2.39, SD 0.41) had significantly lower mean MARS scores. This is in agreement with the findings of a previous review study that found the functionality and usability of apps increased over a 2-year period, but content credibility did not [34]. Therefore, users might need to use their discretion if using outdated apps.

Of the 69 mHealth apps included, *OncoPower, Outcomes4Me Breast Cancer Care, OWise – Breast Cancer Support*, and *War On Cancer* had the highest scores. These apps are available on both Google Play and the App Store, and had high scores for aesthetics, functionality, and engagement. Although *OncoPower* and *War On Cancer* do not target patients with BC and survivors exclusively, they have multiple functions that support patients with other cancer types, such as those related to nutrition and meditation. On the other hand, *Outcomes4Me Breast Cancer Care* and *Owise – Breast Cancer Support* were developed specifically for patients with BC, so these provide evidence-based treatment options and personalized resources based on individuals’ medical records to promote communication with their health care providers. The *War On Cancer* app was developed to promote social networking by patients with cancer and survivors, which may be useful given the importance of social support in cancer care [35-37]. The 4 highest-ranked apps focused mainly on providing personalized care after a diagnosis of BC and on treatment, a time at which patients may struggle due to physical and psychological impairments. Therefore, providing appropriate information on treatment options and lifestyle modification, facilitating medication management, providing psychological support, and tracking PGHD and medical records may ease the burden on patients.

Our results show approximately one-third (n=19, 28%) of the included apps are using individual data, such as PGHD. PGHD, including patient-reported outcomes, provide clinically relevant information obtained outside traditional care settings, and could be useful to improve outcomes and enhance patient-provider communication [38]. Some studies found that effective physician-patient communication improved patient health outcomes [39], as well as BC patients’ depression and quality of life [40]. With the increasing use of wearable devices and advancements in technology, the use of PGHD and established medical screening and surveillance strategies may enhance long-term cancer survivorship at the individual and population levels. Furthermore, these approaches can strengthen the survivor-provider relationship [41]. Despite the benefits of PGHD, there are several barriers to their use, including a lack of technical support in patients’ primary language, the reluctance of clinicians to review PGHD, a lack of access to broadband internet, and concerns related to the confidentiality of personal information [42]. Nevertheless, mobile apps that use medical records and PGHD had the highest MARS scores (4.0 and 3.83, respectively), indicating the willingness of users to use individualized health-related apps.

The large differences in MARS scores among the apps may imply a need to improve the standards used for their approval, and for quality checks at all stages of development (assessment, prototype, content, and evaluation). Some studies strived to evaluate health-related apps by establishing a practical framework based on guidelines by the US Food and Drug Administration and the UK National Health Service [43], or by organizing published studies [44]. This framework was developed by organizing app evaluation questions from 45 previous systematic reviews and verified by the patient advisory panel. It represents the pyramid shape that begins at the bottom from background information, privacy, and security, to evidence based, ease of use, and data integration. If these kinds of evaluations were implemented effectively at the time of app approval, higher-quality apps might be released. Most BC-related apps included in this study provided information or education regarding prevention and survivorship, in line with previous studies [4,26]. However, the mean MARS score of those that provided information at any CCC stage was slightly above 3, which means acceptable but not good enough. Provision of information is important but insufficient to modify multifaceted health behaviors [45]. Additionally, patients with limited health literacy are at a distinct disadvantage during BC treatment in terms of unmet information needs [46]. Moreover, information and educational content that are not based on guidelines or evidence discourage women from consistently using mobile BC apps; this may explain the lower MARS scores on those apps that merely provided information. Furthermore, the MARS scores of apps that targeted patients after a diagnosis of BC, including those that provided survivor support, were higher than those reported previously [26]. Patients who are unconcerned regarding their health while being investigated for cancer may change their attitude toward cancer after their diagnosis [47]. Therefore, such people might search for and use the helpful tools provided by mobile apps.

### Strengths and Limitations

The strengths of our study include using the MARS, an objective tool used to measure app quality. Star ratings and user reviews are also valuable for developers and potential new users because they offer a crowdsourced indicator of the effectiveness and popularity of apps [48]. However, these indicators do not accurately reflect app quality [49]. The majority of public reviews of apps rely on the personal opinions of individuals’ own experience and are thus highly subjective. Therefore, we did not record star ratings in this study. Additionally, a previous study found that MARS scores did not significantly correlate with users’ star ratings [50]. We could not confirm this in our study because of a lack of star ratings and reviews. Therefore, we employed the MARS to assess the quality of the most credible apps. Furthermore, we used these quantitative results to identify the relationship between MARS dimensions and apps’ content.

This study had several limitations. First, paid and inaccessible apps could not be downloaded because of a lack of funding and access. Such apps should be evaluated in future studies for external validity. Second, although the MARS has been widely used and validated previously, we cannot rule out subjectivity due to the nature of evaluations. However, to minimize subjectivity, we confirmed high interrater reliability between the independent raters. Lastly, qualitative measures may be needed to reflect end users’ experience.

### Recommendations for Future Development

The growing numbers of BC-related mHealth apps and of studies on their usefulness allow women to select those most likely to improve their quality of life. We identified certain issues that could be addressed to improve app quality. First, the quality assessment system for the digital platform needs to be improved. Our results identified limitations in certain MARS dimensions, indicating poor app quality. Therefore, a new method should be introduced to validate the MARS that reflects differences between specific diseases and users’ experience [32]. Second, evidence-based content and functions are required. Although the mechanisms underlying the effects of mHealth apps are not known, efforts should be made toward elucidating them by developing a theory-based intervention that is administered via an mHealth app and tested in a clinical trial. Although some feasibility studies and trials have been conducted on the usefulness of PGHD [51,52], further studies of the clinical benefits of mHealth apps are required. The development of a set of core outcomes may be another option to measure their effectiveness and induce behavior change, as has been found by others [53]. Additionally, patient-centered considerations of the design and interface before the development of an app might be helpful to promote behavior change. One study suggested iterative development through a user-centered design approach involving the following 3 phases: analysis, design, and implementation to achieve less fragmented care [54]. This systematic approach could encourage patients, survivors, and health care providers to participate in the development and quality assessment of mobile apps. In sum, the mapping of app content against current BC guidelines and creating adequate evidence would help clinicians have more information about useful content and promote them to recommend using related mobile apps.

### Conclusions

We systematically analyzed 69 BC-related mHealth apps, using literature-based content categories and the MARS for quality assessment. Generally, the quality of the apps was acceptable according to the MARS, but the gap between the highest- and lowest-rated apps was significant. Our findings indicate that the BC-related apps using personalized data were of higher quality than those that merely provided women with information on BC, especially after treatment in the CCC. We also found that apps that had been updated within 1 year and those developed by private companies had higher MARS scores. These findings provide specific criteria for women and clinicians to help them choose the right mobile BC apps for better clinical outcomes.

## Appendix

Multimedia Appendix 1 Content categories for mobile apps related to cancer management.

Multimedia Appendix 2 Full list of included breast cancer apps (n=69).

Multimedia Appendix 3 Content analyses of the 69 breast cancer mobile apps by update year and developer.

Multimedia Appendix 4 Details on the content of the included apps (n=69).

Multimedia Appendix 5 Details on the results of quality assessment of the included apps (n=69).

## Abbreviations

BC: breast cancer
CCC: cancer care continuum
MARS: Mobile App Rating Scale
mHealth: mobile health
PGHD: patient-generated health data
