# Supplementary material for: Mobile Health Apps for Breast Cancer: Content Analysis and Quality Assessment
Source: JMIR Mhealth Uhealth. 2023 Feb 23;11:e43522. doi: 10.2196/43522 (PMC9999256; doi:10.2196/43522)
Supplement: Multimedia Appendix 1 [file mhealth_v11i1e43522_app1.docx]

Multimedia Appendix 1. Content categories for mobile applications related to cancer management

| Cancer Control Continuum | Charbonneau et al. 2020 | Definition | Content | Definition |
| --- | --- | --- | --- | --- |
| Etiology | Disease and treatment information | Information about disease or treatment options | Information on risk factors | Provides information regarding risk factors and biological processes of BC |
| and |  |  |  |  |
|  |  |  |  |  |
| Prevention | Prevention | Information and practical tools to avoid cancer, including recurrence | Risk prediction | Provides a tool to predict BC risk |
|  |  |  |  |  |
|  | Educational | General information and tools to raise public awareness of cancer as a societal problem; includes descriptions and statistics | Education for BC prevention and risk factors | Provides facts and knowledge related to BC prevention |
|  |  |  |  |  |
| Detection | Early detection | Information and tools to assist in the identification of cancer before the emergence of symptoms or signs (i.e., breast self-examinations and mole/skin check apps) | Self-examination guidance | Sets alarms and provides instructions for breast self-exam |
|  |  |  |  |  |
|  | Educational | General information and tools to raise public awareness of cancer as a societal problem; includes descriptions and statistics | Education for early detection | Provides education for early BC detection, including self-exam, breast exam, and mammography |
|  |  |  |  |  |
|  | Support | Access to peer or professional assistance, including information and photograph sharing | Connection to professionals | Connects to professionals at nearby medical centers for early BC detection (e.g., using mammograms) |
| Diagnosis |  |  | Not applicable |  |
| and |  |  |  |  |
| Treatment | Disease and treatment information | Information about disease or treatment options | Information on BC treatment | Provides information about symptoms, treatments, new advancements in breast cancer treatment, and side effects related to BC treatment |
|  |  |  |  |  |
|  | Disease management | Information and practical tools to deal with medical, behavioral, or emotional aspects of cancer | Patient-generated health data | Tracks lifestyle factors (e.g., exercise, sleep, diet, symptoms, and menstruation) |
|  |  |  | Medical records | Tracks personal medical records |
|  |  |  | Medication management | Provides notifications regarding medication time and records, and information on different BC medications |
|  |  |  |  |  |
|  | Support | Access to peer or professional assistance, including information and photograph sharing | Consultation with a physician | Provides a point of contact with a physician for consultation regarding treatment |
|  |  |  | Tracks appointments | Tracks appointments (e.g., consultations, treatments, and investigations) with healthcare providers (personal records) |
|  |  |  | Shares medical records | Shares medical records with health professionals |
| Survivorship | Disease management | Information about disease or treatment options | Information on post-treatment care and prevention of cancer recurrence | Provides information on post-treatment monitoring and medical care, and signs of need for assistance |
|  |  |  |  |  |
|  | Educational | Information and practical tools to deal with medical, behavioral, or emotional aspects of cancer | Post-treatment education | Provides knowledge about post-treatment care, diet, exercise, mental care, and prevention of recurrence |
|  |  |  |  |  |
|  | Support | Access to peer or professional assistance, including information and photograph sharing | Consultation with an expert | Provides a point of contact for consultations with health professionals (i.e., about diet and exercise) via the app |
|  |  |  | Psychological support | Supports psychological aspects of BC patients (e.g., mindfulness and meditation) |
|  |  |  | Community | Aids communication with other patients who have the same condition |
|  |  |  | Communication with family and caregiver | Aids communication with other patients and caregivers/family |
|  |  |  |  |  |
|  | Fundraising | Tools to attract financial resources | Fundraising | Encourages fundraising for BC survivors |

BC: breast cancer
