# Supplementary material for: Mobile Health Apps for Breast Cancer: Content Analysis and Quality Assessment
Source: JMIR Mhealth Uhealth. 2023 Feb 23;11:e43522. doi: 10.2196/43522 (PMC9999256; doi:10.2196/43522)
Supplement: Multimedia Appendix 2 [file mhealth_v11i1e43522_app2.docx]

Multimedia Appendix 2. Full list of included breast cancer apps (n=69).

| Platform | Index for apps | Title | Country | Updated within (year) | Developer |
| --- | --- | --- | --- | --- | --- |
| Android | A1 | Comcomi | South Korea | 3 | I |
|  | A2 | Pink Touch | South Korea | 1 | C |
|  | A3 | BRAVO | South Korea | 1 | C |
|  | A4 | Breast Cancer by Second Doctor | South Korea | 1 | C |
|  | A5 | The BAPS App Wales | UK | 3 | C |
|  | A6 | Breast Cancer Risk Assessment | USA | 3 | C |
|  | A7 | Breast Cancer Questions | NA | 3 | I |
|  | A8 | ABCs of Breast Health | India | 3 | P |
|  | A9 | CANScreen By Mathew Varghese V | India | 3 | C |
|  | A10 | Breast Cancer Stages, Signs, Food and Meal Plan | USA | 3 | C |
|  | A11 | Cancer Guide | India | 3 | C |
|  | A12 | Breast Cancer | USA | 3 | I |
|  | A13 | Pinky Promise-Al Noor | India | 3 | C |
|  | A14 | Breast Care: Helpful Tips To Keep Breasts Healthy | NA | 3 | I |
|  | A15 | Breast Cancer Info | NA | 3 | I |
|  | A16 | BREAST TEST | Slovenia | 2 | C |
|  | A17 | CanApp by Can Protect Foundation | India | 2 | P |
|  | A18 | Becca - Breast Cancer Support | UK | 1 | P |
|  | A19 | MFHP: Cancer | USA | 1 | P |
|  | A20 | Pink Decoder | Bangladesh | 1 | I |
|  | A21 | Centafit: Health Check, Screening, Life Expectancy | Indonesia | 1 | C |
|  | A22 | PinkShield | Arab Emirates | 1 | P |
|  | A23 | Know Your Lemons Breast Health App | USA | 1 | P |
|  | A24 | Breast Cancer Survival Guide for Patients | NA | 1 | I |
|  | A25 | Breast Cancer | NA | 1 | C |
|  | A26 | breastcare - breast awareness | Germany | 1 | P |
|  | A27 | Oncopower | USA | 1 | C |
|  | A28 | Wave: Health & Symptom Tracker | USA | 1 | C |
|  | A29 | Bezzy BC | USA | 1 | C |
|  | A30 | Outcomes4Me Breast Cancer Care | USA | 1 | C |
|  | A31 | Breast Cancer | NA | 3 | I |
|  | A32 | Beat Cancer | India | 3 | C |
|  | A33 | NIH: Breast Cancer | USA | 2 | C |
|  | A34 | Symptoms of Breast Cancer | USA | 2 | C |
|  | A35 | OWise - Breast Cancer Support | USA | 2 | C |
|  | A36 | DearMamma | Switzerland | 2 | C |
|  | A37 | Breast Advocate | USA | 1 | C |
|  | A38 | BCare: Examine Yourself | USA | 1 | P |
|  | A39 | BELONG Beating Cancer Together | USA | 1 | C |
|  | A40 | War On Cancer: Join Today | USA | 1 | C |
|  | A41 | My CareCrew - Cancer Support | USA | 1 | C |
| iOS | I1 | Pink Touch | South Korea | 1 | C |
|  | I2 | WEPEACH | South Korea | 1 | C |
|  | I3 | Breast Cancer by Second Doctor | South Korea | 1 | C |
|  | I4 | Counterpart Navigator | Australia | 3 | C |
|  | I5 | Pink Pakistan | Pakistan | 2 | P |
|  | I6 | The Cancer App | UK | 1 | C |
|  | I7 | Feel For Your Life | USA | 1 | C |
|  | I8 | Breast Aware - BCI | Ireland | 1 | C |
|  | I9 | Becca - Breast Cancer Support | UK | 1 | P |
|  | I10 | ACS Reach | USA | 1 | P |
|  | I11 | Pinkypromise-Alnoor | India | 1 | C |
|  | I12 | PinkShield | Arab Emirates | 1 | P |
|  | I13 | Triple Negative Breast Cancer | USA | 1 | C |
|  | I14 | Wave: Health & Symptom Tracker | USA | 1 | C |
|  | I15 | breastcare - breast awareness | Germany | 1 | P |
|  | I16 | Boot Out Breast Cancer | USA | 1 | P |
|  | I17 | OncoPower | USA | 1 | C |
|  | I18 | Outcomes4Me Breast Cancer Care | USA | 1 | C |
|  | I19 | My Journey (BCNA) | Australia | 2 | P |
|  | I20 | DearMamma | Switzerland | 1 | P |
|  | I21 | BCare: Examine Yourself | USA | 1 | C |
|  | I22 | Cancer Symptom Tracker | USA | 1 | C |
|  | I23 | Breast Cancer Manager | USA | 1 | C |
|  | I24 | OWise - Breast Cancer Support | USA | 1 | C |
|  | I25 | War On Cancer - Social App | USA | 1 | C |
|  | I26 | My CareCrew - Cancer Support | USA | 1 | C |
|  | I27 | Know Your Lemons Breast Check | USA | 1 | P |
|  | I28 | Keep A Breast | USA | 1 | P |
| NA: not assessable, I: individual, C: commercial organization, P: public institution | | | | | |
