# Supplementary material for: Mobile Health Apps for Breast Cancer: Content Analysis and Quality Assessment
Source: JMIR Mhealth Uhealth. 2023 Feb 23;11:e43522. doi: 10.2196/43522 (PMC9999256; doi:10.2196/43522)
Supplement: Multimedia Appendix 3 [file mhealth_v11i1e43522_app3.docx]

Multimedia Appendix 3. Content analyses of the 69 breast cancer mobile apps by update year and developer.

| Cancer Control Continuum | Content | Updated within | | | | | |  | Developer | | | | | |  | Total (n=69) | |
| --- | --- | --- | --- | --- | --- | --- | --- | --- | --- | --- | --- | --- | --- | --- | --- | --- | --- |
|  |  | 1 year (n=46) | | 2 years (n=8) | | 3 years (n=15) | |  | Individual (n=8) | | Commercial organization (n=43) | | Public institution (n=18) | |  | n | % |
|  |  | n | % | n | % | n | % |  | n | % | n | % | n | % |  |  |  |
| Etiology and prevention | Information on BC | 15 | 32.6 | 5 | 62.5 | 8 | 53.3 |  | 5 | 62.5 | 14 | 32.6 | 9 | 50.0 |  | 28 | 40.6 |
|  | Risk prediction | 7 | 15.2 | 0 | 0.0 | 3 | 20.0 |  | 2 | 25.0 | 4 | 9.3 | 4 | 22.2 |  | 10 | 14.5 |
|  | Education for prevention & risk factors for BC | 13 | 28.3 | 2 | 25.0 | 7 | 46.7 |  | 5 | 62.5 | 9 | 20.9 | 8 | 44.4 |  | 22 | 31.9 |
| Detection | Guidance for early detection | 22 | 47.8 | 6 | 75.0 | 6 | 40.0 |  | 4 | 50.0 | 17 | 39.5 | 13 | 72.2 |  | 34 | 49.3 |
|  | Connection to professionals | 8 | 17.4 | 1 | 12.5 | 1 | 6.7 |  | 1 | 12.5 | 1 | 2.3 | 8 | 44.4 |  | 10 | 14.5 |
| Diagnosis and treatment | Information on BC treatment | 15 | 32.6 | 4 | 50.0 | 8 | 53.3 |  | 5 | 62.5 | 18 | 41.9 | 4 | 22.2 |  | 27 | 39.1 |
|  | Patient-generated health data (PGHD) | 16 | 34.8 | 3 | 37.5 | 0 | 0.0 |  | 0 | 0.0 | 16 | 37.2 | 3 | 16.7 |  | 19 | 27.5 |
|  | Medical records | 5 | 10.9 | 0 | 0.0 | 1 | 6.7 |  | 1 | 12.5 | 5 | 11.6 | 0 | 0.0 |  | 6 | 8.7 |
|  | Medication management | 14 | 30.4 | 1 | 12.5 | 1 | 6.7 |  | 1 | 12.5 | 15 | 34.9 | 0 | 0.0 |  | 16 | 23.2 |
|  | Consultation by a physician | 4 | 8.7 | 0 | 0.0 | 0 | 0.0 |  | 0 | 0.0 | 4 | 9.3 | 0 | 0.0 |  | 4 | 5.8 |
|  | Tracking appointment | 6 | 13.0 | 2 | 25.0 | 1 | 6.7 |  | 1 | 12.5 | 7 | 16.3 | 1 | 5.6 |  | 9 | 13.0 |
|  | Participation in decision making | 7 | 15.2 | 1 | 12.5 | 1 | 6.7 |  | 1 | 12.5 | 4 | 9.3 | 4 | 22.2 |  | 9 | 13.0 |
| Survivorship | Information on post-treatment care & prevention of recurrence | 8 | 17.4 | 3 | 37.5 | 3 | 20.0 |  | 2 | 25.0 | 8 | 18.6 | 4 | 22.2 |  | 14 | 20.3 |
|  | Education for lifestyle modification | 15 | 32.6 | 2 | 25.0 | 5 | 33.3 |  | 1 | 12.5 | 17 | 39.5 | 4 | 22.2 |  | 23 | 33.3 |
|  | Consultation by an expert | 10 | 21.7 | 0 | 0.0 | 0 | 0.0 |  | 0 | 0.0 | 8 | 18.6 | 2 | 11.1 |  | 10 | 14.5 |
|  | Psychological support | 16 | 34.8 | 1 | 12.5 | 0 | 0.0 |  | 0 | 0.0 | 11 | 25.6 | 6 | 33.3 |  | 17 | 24.6 |
|  | Community | 12 | 26.1 | 1 | 12.5 | 0 | 0.0 |  | 1 | 12.5 | 10 | 23.3 | 2 | 11.1 |  | 13 | 18.8 |
|  | Sharing information with family and caregiver | 6 | 13.0 | 1 | 12.5 | 0 | 0.0 |  | 0 | 0.0 | 6 | 14.0 | 1 | 5.6 |  | 7 | 10.1 |
|  | Fundraising | 7 | 15.2 | 1 | 12.5 | 0 | 0.0 |  | 0 | 0.0 | 2 | 4.7 | 6 | 33.3 |  | 8 | 11.6 |
