# Supplementary material for: Mobile Health Apps for Breast Cancer: Content Analysis and Quality Assessment
Source: JMIR Mhealth Uhealth. 2023 Feb 23;11:e43522. doi: 10.2196/43522 (PMC9999256; doi:10.2196/43522)
Supplement: Multimedia Appendix 4 [file mhealth_v11i1e43522_app4.docx]

Multimedia Appendix 4. Details on content of included apps (n=69).

| Platform | Index for apps | Content | | | | | | | | | | | | | | | | | | |
| --- | --- | --- | --- | --- | --- | --- | --- | --- | --- | --- | --- | --- | --- | --- | --- | --- | --- | --- | --- | --- |
|  |  | Etiology and prevention | | | Detection | | Diagnosis and treatment | | | | | | | Survivorship | | | | | | |
|  |  | Information on BC | Risk assessment or prediction | Education for prevention and risk factors | Guidance for early detection | Connection to professionals | Information on BC treatment | PGHD | Medical records | Medication management | Consultation by a physician | Tracking appointments | Participation in decision making | Information on post treatment | Education for lifestyle modification | Consultation by an expert | Psychological support | Community | Sharing information with family and caregiver | Fundraising |
| Android | A1 |  |  |  |  |  |  |  | x | x |  | x |  |  |  |  |  |  |  |  |
|  | A2 | x |  | x | x |  |  |  |  |  |  |  |  |  |  |  |  |  |  |  |
|  | A3 | x |  | x | x |  | x | x |  | x |  |  |  | x | x |  | x |  |  |  |
|  | A4 |  |  |  |  |  |  | x |  | x |  |  |  |  | x | x |  |  |  |  |
|  | A5 |  |  |  |  |  |  |  |  |  |  |  |  |  | x |  |  |  |  |  |
|  | A6 |  | x |  |  |  |  |  |  |  |  |  |  |  |  |  |  |  |  |  |
|  | A7 | x |  |  |  |  | x |  |  |  |  |  | x | x |  |  |  |  |  |  |
|  | A8 | x |  |  | x |  | x |  |  |  |  |  |  | x | x |  |  |  |  |  |
|  | A9 | x |  | x | x |  |  |  |  |  |  |  |  |  |  |  |  |  |  |  |
|  | A10 | x |  | x |  |  |  |  |  |  |  |  |  |  | x |  |  |  |  |  |
|  | A11 |  | x |  |  | x | x |  |  |  |  |  |  |  |  |  |  |  |  |  |
|  | A12 | x | x | x |  |  | x |  |  |  |  |  |  |  |  |  |  |  |  |  |
|  | A13 |  |  |  | x |  |  |  |  |  |  |  |  |  |  |  |  |  |  |  |
|  | A14 |  |  | x | x |  |  |  |  |  |  |  |  |  |  |  |  |  |  |  |
|  | A15 | x |  | x |  |  | x |  |  |  |  |  |  |  |  |  |  |  |  |  |
|  | A16 |  |  |  | x |  |  |  |  |  |  |  |  |  |  |  |  |  |  |  |
|  | A17 | x |  |  | x |  |  | x |  |  |  | x |  |  |  |  |  |  |  | x |
|  | A18 |  |  |  |  |  |  |  |  |  |  |  |  | x |  | x | x |  |  |  |
|  | A19 | x | x |  |  |  |  |  |  |  |  |  |  |  |  |  |  |  | x |  |
|  | A20 | x | x |  | x | x |  |  |  |  |  |  |  |  |  |  |  | x |  |  |
|  | A21 |  | x |  | x |  |  |  |  |  |  |  |  |  |  |  |  |  | x |  |
|  | A22 | x |  | x | x | x | x |  |  |  |  |  | x |  | x |  | x |  |  |  |
|  | A23 |  | x | x | x | x |  |  |  |  |  |  | x |  |  |  |  |  |  | x |
|  | A24 |  |  | x | x |  | x |  |  |  |  |  |  | x | x |  |  |  |  |  |
|  | A25 | x |  | x | x |  |  |  |  |  |  |  |  |  |  |  |  |  |  |  |
|  | A26 |  |  | x | x | x |  |  |  |  |  |  |  |  |  |  |  |  |  | x |
|  | A27 |  |  |  |  |  | x | x | x | x | x |  |  | x | x | x | x | x |  |  |
|  | A28 |  |  |  |  |  |  | x |  | x |  |  |  |  |  |  |  |  |  |  |
|  | A29 |  |  |  |  |  |  |  |  |  |  |  |  |  | x |  | x | x |  |  |
|  | A30 | x |  |  |  |  | x | x | x | x |  |  |  |  | x | x | x |  |  |  |
|  | A31 | x |  | x | x |  | x |  |  |  |  |  |  |  |  |  |  |  |  |  |
|  | A32 | x |  | x | x |  | x |  |  |  |  |  |  |  | x |  |  |  |  |  |
|  | A33 | x |  | x | x |  | x |  |  |  |  |  |  | x | x |  |  |  |  |  |
|  | A34 | x |  |  | x |  | x |  |  |  |  |  |  | x |  |  |  |  |  |  |
|  | A35 |  |  |  |  |  | x | x |  | x |  | x | x |  |  |  |  |  | x |  |
|  | A36 |  |  |  | x |  |  |  |  |  |  |  |  |  |  |  |  |  |  |  |
|  | A37 |  |  |  |  |  | x |  |  |  | x |  | x |  | x | x |  | x |  |  |
|  | A38 | x | x | x | x |  |  |  |  |  |  |  |  |  |  |  |  |  |  |  |
|  | A39 |  |  |  |  |  | x |  | x |  | x | x |  |  | x | x |  | x | x |  |
|  | A40 |  |  |  |  |  |  |  |  |  |  |  |  |  | x |  | x | x |  |  |
|  | A41 |  |  |  |  |  |  | x |  |  |  |  |  |  |  |  |  | x | x |  |
| iOS | I1 | x |  | x | x |  |  |  |  |  |  |  |  |  |  |  |  |  |  |  |
|  | I2 |  |  |  | x |  |  | x |  | x |  | x |  | x |  |  | x | x |  |  |
|  | I3 |  |  |  |  |  |  | x |  | x |  |  |  |  | x | x |  |  |  |  |
|  | I4 |  |  |  |  |  | x |  |  |  |  |  |  | x | x |  |  |  |  |  |
|  | I5 | x |  | x | x | x |  |  |  |  |  |  |  |  |  |  |  | x |  |  |
|  | I6 | x |  |  |  |  | x |  |  | x |  | x |  |  |  |  | x |  |  |  |
|  | I7 |  |  |  | x |  | x |  |  |  |  |  |  |  |  |  |  |  |  | x |
|  | I8 |  |  |  | x |  |  |  |  |  |  |  |  |  |  |  |  |  |  | x |
|  | I9 |  |  |  |  |  |  |  |  |  |  |  |  | x |  | x | x |  |  |  |
|  | I10 |  |  |  |  |  |  |  |  |  |  |  |  |  |  |  | x | x |  |  |
|  | I11 |  |  |  | x |  |  |  |  |  |  |  |  |  |  |  |  |  |  |  |
|  | I12 | x |  | x | x | x | x |  |  |  |  |  | x |  | x |  | x |  |  |  |
|  | I13 | x |  |  |  |  | x |  |  |  |  |  |  |  |  |  |  |  |  |  |
|  | I14 |  |  |  |  |  |  | x |  | x |  |  |  |  |  |  |  |  |  |  |
|  | I15 |  |  | x | x | x |  |  |  |  |  |  |  |  |  |  |  |  |  | x |
|  | I16 | x |  |  | x |  |  | x |  |  |  |  |  |  |  |  |  |  |  | x |
|  | I17 |  |  |  |  |  | x | x | x | x | x |  |  | x | x | x | x | x |  |  |
|  | I18 | x |  |  |  |  | x | x | x | x |  |  |  |  | x | x | x |  |  |  |
|  | I19 | x |  |  |  |  | x | x |  |  |  |  |  | x | x |  | x |  |  |  |
|  | I20 |  |  |  | x |  |  |  |  |  |  |  |  |  |  |  |  |  |  |  |
|  | I21 | x | x | x | x |  |  |  |  |  |  |  |  |  |  |  |  |  |  |  |
|  | I22 |  |  |  |  |  |  | x |  | x |  | x |  | x |  |  | x |  |  |  |
|  | I23 |  |  |  |  |  | x | x |  | x |  | x | x |  |  |  |  |  |  |  |
|  | I24 |  |  |  |  |  | x | x |  | x |  | x | x |  |  |  |  |  | x |  |
|  | I25 |  |  |  |  |  |  |  |  |  |  |  |  |  | x |  | x | x |  |  |
|  | I26 |  |  |  |  |  |  | x |  |  |  |  |  |  |  |  |  | x | x |  |
|  | I27 |  | x | x | x | x |  |  |  |  |  |  | x |  |  |  |  |  |  | x |
|  | I28 |  |  |  | x | x |  |  |  |  |  |  |  |  |  |  |  |  |  |  |
