# Supplementary material for: Mobile Health Apps for Breast Cancer: Content Analysis and Quality Assessment
Source: JMIR Mhealth Uhealth. 2023 Feb 23;11:e43522. doi: 10.2196/43522 (PMC9999256; doi:10.2196/43522)
Supplement: Multimedia Appendix 5 [file mhealth_v11i1e43522_app5.docx]

Multimedia Appendix 5. Details on results of quality assessment of included apps (n=69).

| Platform | Index for apps | Country | Updated within (year) | Developer | MARS | | | | | |
| --- | --- | --- | --- | --- | --- | --- | --- | --- | --- | --- |
|  |  |  |  |  | Total mean | A. Engagement | B. Functionality | C. Aesthetics | D. Information | E. App subjective quality |
| Android | A1 | South Korea | 3 | I | 3.1 | 3.2 | 3.0 | 3.5 | 3.5 | 1.9 |
|  | A2 | South Korea | 1 | C | 3.9 | 3.8 | 4.6 | 5.0 | 3.9 | 2.4 |
|  | A3 | South Korea | 1 | C | 3.7 | 3.6 | 4.6 | 4.5 | 3.8 | 2.4 |
|  | A4 | South Korea | 1 | C | 4.0 | 4.1 | 4.3 | 4.7 | 4.3 | 2.5 |
|  | A5 | UK | 3 | C | 3.3 | 2.8 | 4.8 | 3.5 | 3.4 | 2.4 |
|  | A6 | USA | 3 | C | 2.7 | 2.0 | 4.0 | 3.0 | 3.4 | 1.5 |
|  | A7 | NA | 3 | I | 2.9 | 1.8 | 3.9 | 3.2 | 4.0 | 1.5 |
|  | A8 | India | 3 | P | 2.8 | 2.3 | 4.5 | 1.8 | 3.4 | 1.5 |
|  | A9 | India | 3 | C | 2.9 | 2.2 | 4.5 | 2.3 | 3.3 | 1.8 |
|  | A10 | USA | 3 | C | 2.7 | 1.4 | 4.4 | 3.5 | 3.1 | 1.4 |
|  | A11 | India | 3 | C | 2.3 | 1.6 | 4.1 | 2.0 | 2.4 | 1.4 |
|  | A12 | USA | 3 | I | 1.9 | 1.2 | 4.4 | 1.5 | 1.7 | 1.0 |
|  | A13 | India | 3 | C | 2.1 | 1.4 | 3.5 | 1.7 | 2.8 | 1.0 |
|  | A14 | NA | 3 | I | 2.2 | 1.6 | 4.1 | 1.3 | 2.6 | 1.1 |
|  | A15 | NA | 3 | I | 2.2 | 1.3 | 4.6 | 2.5 | 1.8 | 1.1 |
|  | A16 | Slovenia | 2 | C | 3.4 | 3.0 | 4.3 | 3.8 | 3.6 | 2.3 |
|  | A17 | India | 2 | P | 3.0 | 2.5 | 3.9 | 2.8 | 3.8 | 1.6 |
|  | A18 | UK | 1 | P | 3.5 | 2.8 | 4.3 | 4.5 | 3.8 | 2.3 |
|  | A19 | USA | 1 | P | 3.5 | 2.9 | 4.5 | 4.2 | 4.1 | 2.0 |
|  | A20 | Bangladesh | 1 | I | 2.3 | 2.0 | 3.8 | 1.8 | 2.5 | 1.3 |
|  | A21 | Indonesia | 1 | C | 4.0 | 4.0 | 4.4 | 4.3 | 4.2 | 2.9 |
|  | A22 | Arab Emirates | 1 | P | 3.4 | 2.3 | 4.6 | 3.8 | 4.2 | 2.3 |
|  | A23 | USA | 1 | P | 3.6 | 3.7 | 3.8 | 4.3 | 3.9 | 2.4 |
|  | A24 | NA | 1 | I | 2.4 | 1.8 | 3.9 | 1.7 | 3.1 | 1.4 |
|  | A25 | NA | 1 | C | 2.0 | 1.4 | 4.5 | 1.5 | 1.8 | 1.0 |
|  | A26 | Germany | 1 | P | 3.6 | 3.2 | 4.0 | 4.7 | 4.1 | 2.5 |
|  | A27 | USA | 1 | C | 4.0 | 4.1 | 4.4 | 4.5 | 3.8 | 3.4 |
|  | A28 | USA | 1 | C | 4.1 | 3.7 | 4.8 | 4.8 | 3.8 | 3.5 |
|  | A29 | USA | 1 | C | 3.8 | 4.0 | 4.3 | 4.7 | 4.0 | 2.3 |
|  | A30 | USA | 1 | C | 4.4 | 4.4 | 4.3 | 4.8 | 4.5 | 4.1 |
|  | A31 | NA | 3 | I | 2.1 | 1.6 | 3.9 | 1.5 | 2.2 | 1.1 |
|  | A32 | India | 3 | C | 2.5 | 1.4 | 4.0 | 2.8 | 3.0 | 1.4 |
|  | A33 | USA | 2 | C | 2.6 | 1.8 | 4.8 | 2.8 | 2.7 | 1.3 |
|  | A34 | USA | 2 | C | 2.0 | 1.2 | 4.6 | 1.3 | 2.0 | 1.0 |
|  | A35 | USA | 2 | C | 4.2 | 3.7 | 4.5 | 4.5 | 4.1 | 3.9 |
|  | A36 | Switzerland | 2 | C | 3.6 | 3.3 | 4.6 | 4.0 | 3.8 | 2.3 |
|  | A37 | USA | 1 | C | 3.4 | 3.2 | 3.8 | 3.7 | 4.1 | 2.4 |
|  | A38 | USA | 1 | P | 3.8 | 3.3 | 4.9 | 5.0 | 3.8 | 2.4 |
|  | A39 | USA | 1 | C | 3.8 | 3.5 | 4.8 | 4.8 | 3.7 | 2.9 |
|  | A40 | USA | 1 | C | 4.0 | 3.6 | 4.8 | 5.0 | 4.1 | 2.9 |
|  | A41 | USA | 1 | C | 3.3 | 3.0 | 3.9 | 4.5 | 3.5 | 2.1 |
| iOS | I1 | South Korea | 1 | C | 3.9 | 3.3 | 4.8 | 5.0 | 4.3 | 2.5 |
|  | I2 | South Korea | 1 | C | 4.0 | 3.9 | 4.4 | 4.2 | 4.1 | 3.3 |
|  | I3 | South Korea | 1 | C | 4.0 | 4.1 | 4.1 | 4.8 | 4.1 | 2.9 |
|  | I4 | Australia | 3 | C | 2.4 | 2.0 | 3.9 | 2.5 | 2.6 | 1.3 |
|  | I5 | Pakistan | 2 | P | 3.0 | 2.4 | 4.8 | 3.7 | 3.1 | 1.5 |
|  | I6 | UK | 1 | C | 3.3 | 3.1 | 4.3 | 2.8 | 4.0 | 2.1 |
|  | I7 | USA | 1 | C | 3.0 | 2.7 | 4.1 | 3.2 | 2.9 | 2.0 |
|  | I8 | Ireland | 1 | C | 3.2 | 2.6 | 4.8 | 4.5 | 3.0 | 1.8 |
|  | I9 | UK | 1 | P | 3.5 | 3.0 | 4.5 | 4.7 | 3.7 | 2.1 |
|  | I10 | USA | 1 | P | 3.4 | 3.0 | 4.0 | 4.0 | 4.2 | 2.1 |
|  | I11 | India | 1 | C | 3.1 | 2.6 | 5.0 | 4.2 | 2.7 | 1.6 |
|  | I12 | Arab Emirates | 1 | P | 3.4 | 2.2 | 4.8 | 3.7 | 4.1 | 2.4 |
|  | I13 | USA | 1 | C | 3.1 | 2.9 | 4.1 | 2.8 | 3.6 | 1.8 |
|  | I14 | USA | 1 | C | 4.2 | 3.6 | 4.9 | 5.0 | 4.0 | 3.5 |
|  | I15 | Germany | 1 | P | 3.7 | 3.5 | 4.6 | 4.5 | 3.8 | 2.1 |
|  | I16 | USA | 1 | P | 3.3 | 2.9 | 4.8 | 3.8 | 3.2 | 1.8 |
|  | I17 | USA | 1 | C | 4.2 | 4.1 | 4.6 | 4.8 | 4.0 | 3.8 |
|  | I18 | USA | 1 | C | 4.5 | 4.4 | 4.8 | 5.0 | 4.7 | 3.9 |
|  | I19 | Australia | 2 | P | 3.6 | 3.5 | 4.3 | 4.5 | 3.6 | 2.5 |
|  | I20 | Switzerland | 1 | P | 3.5 | 3.1 | 4.8 | 4.0 | 3.8 | 2.1 |
|  | I21 | USA | 1 | C | 3.6 | 2.8 | 4.9 | 4.7 | 3.5 | 2.5 |
|  | I22 | USA | 1 | C | 4.1 | 4.2 | 4.5 | 4.3 | 4.5 | 3.1 |
|  | I23 | USA | 1 | C | 2.6 | 1.6 | 3.3 | 3.0 | 3.6 | 1.8 |
|  | I24 | USA | 1 | C | 4.1 | 4.0 | 4.4 | 5.0 | 3.9 | 3.5 |
|  | I25 | USA | 1 | C | 4.0 | 3.8 | 5.0 | 4.7 | 3.7 | 3.0 |
|  | I26 | USA | 1 | C | 3.5 | 3.2 | 4.3 | 4.8 | 3.8 | 2.1 |
|  | I27 | USA | 1 | P | 3.7 | 3.8 | 4.3 | 4.7 | 3.7 | 2.5 |
|  | I28 | USA | 1 | P | 3.8 | 3.5 | 5.0 | 4.8 | 3.6 | 2.4 |
| NA: not assessable, I: individual, C: commercial organization, P: public institution | | | | | | | | | | |
